# Aqueous and Methanol Extracts of *Paullinia pinnata* (Sapindaceae) Improve Monosodium Urate-Induced Gouty Arthritis in Rat: Analgesic, Anti-Inflammatory, and Antioxidant Effects

**DOI:** 10.1155/2019/5946291

**Published:** 2019-12-05

**Authors:** Pius Pum Tseuguem, Télesphore Benoît Nguelefack, Basile Ngnanmegne Piégang, Sorelle Mbankou Ngassam

**Affiliations:** Research Unit of Neuro-Inflammatory and Cardiovascular Pharmacology, Laboratory of Animal Physiology and Phytopharmacology, University of Dschang, P.O. Box 67, Dschang, Cameroon

## Abstract

The profound modification of lifestyle and food habits has led to an important increase in the prevalence of gout. Unfortunately, there are current unmet needs for the treatment of this disease, prompting the search for new alternatives. *Paullinia pinnata* is a plant used to treat various diseases including arthritis. The present work aimed to investigate the antigouty activities of the aqueous (AEPP) and methanolic (MEPP) extracts of *P. pinnata* as well as their in vivo antioxidant properties. The gouty arthritis was induced by injecting 50 *μ*l of monosodium urate (MSU, 100 mg/ml) in the left hind ankle of rats. *P. pinnata* extracts were administered orally at the doses of 100 and 200 mg/kg/day for 6 days, starting 24 h after MSU injection. Allopurinol 5 mg/kg/day was used as reference drug. Inflammation and hyperalgesia were daily monitored from 24 hours after treatment initiation and for the 6 consecutive days. Myeloperoxidase (MPO) quantification was done in collected synovial fluid. Nitrite oxide (NO), malondialdehyde (MDA), and superoxide dismutase (SOD) were evaluated in the spinal cord and the brain. The serum content of SOD was additionally quantified. AEPP and MEPP significantly (*p* < 0.001) reduce MSU-induced inflammation (22.41% to 93.65%) and hyperalgesia (33.33% to 64.44%) in both ankle and paw. AEPP and MEPP significantly (*p* < 0.001) reduce synovial MPO production with the percentage ranging from 76.30% to 85.19%. AEPP and MEPP significantly (*p* < 0.001) reduce serum, spinal, left and right hemispheres NO, and MDA and increase the SOD activity (*p* < 0.001). *P. pinnata* leaf extracts possess potent curative effects against MSU-induced gouty arthritis that combines analgesic, anti-inflammatory, and antioxidant activities. These findings support the use of *P. pinnata* leaves extracts in the treatment of gouty arthritis and further present the plant as a potent source of efficient antigouty medicine.

## 1. Introduction

Gouty arthritis is a painful inflammatory disease, characterized by a swollen and painful joint which usually extends to the surrounding tissue. Gouty arthritis usually occurs following hyperuricemia, identified as the major risk factor [1, 2]. This joint disorder is characterized by the precipitation, deposition, and crystallization of monosodium urate in the joints, leading to intense inflammation and painful process [3]. The gout prevalence ranges between 4 and 6.8% [4, 5]. Its incidence is constantly rising, and it is associated with many comorbidities. In the development of the pathology, the deleterious effects of urate are primarily due to its capacity to trigger the production of reactive oxygen species (ROS), thus promoting the oxidative status in gouty arthritis patients. Concordantly, oxidative stress status has been demonstrated in gouty arthritis [6].

The timeline treatment against gout focuses on the treatment of inflammation and the reduction of hyperuricemia. Many agents, including allopurinol, febuxostat, colchicine, nonsteroidal anti-inflammatory drugs, corticosteroids, and more recently interleukin (IL)-1 inhibitors, have been developed and have demonstrated efficiency in gout treatment. Nevertheless, they have some limitations among which are little effect on acute gout attacks, contraindications with the comorbidities, and drugs' side effects, which include fever, skin rashes, allergic reactions, hepatitis, nephropathy, and gastrointestinal toxicity/bleeding [7–9]. There is, therefore, an important need for more effective new therapeutics with low side effects.

One of the most reliable sources of new compounds is made of medicinal plants. They have gained more interest these last decades and are now intensively used by industries for the production of phytopharmaceuticals and nutraceuticals.

The search for new natural drugs with antigouty effects, combining anti-inflammatory, analgesic, and antioxidant properties, is the focus of this manuscript. *Paullinia pinnata* Linn is a creeper plant (Sapindaceae) widely distributed in the west and the center regions of Cameroon. It is empirically used for the treatment of infection and painful and inflammatory diseases. The leaves decoction is used in the East Africa region, for the treatment of snake bites [10]. Phytochemical studies of this plant extract revealed the presence of phenols [11], flavonoids, triterpene, saponins and tannins [12], steroids, steroidal glycosides, and cerebroside and ceramide [13]. Compounds such as 2-*O*-methyl-L-chiro-inositol, *β*-sitosterol, and friedelin have been isolated from *P. pinnata* [14]. Previous pharmacological studies demonstrated the antimicrobial activities and protective effects against CCl4-induced hepatic damage and oxidative stress [14, 15]. Our recent studies showed that the aqueous and methanol extracts from the leaves of *P. pinnata* possess potent preventive effects on rheumatoid monoarthritis [16]. But whether these plant extracts can cure gouty arthritis is unknown. The present study was undertaken to evaluate the curative effect of the aqueous and methanol extracts of *P. pinnata* on the MSU-induced gouty arthritis in Wistar rats.

## 2. Materials and Methods

### 2.1. Plant Material and Preparation of Extracts

The fresh leaves of *P. pinnata* were harvested at Poumougne in the Koung-khi Division (West Region, Cameroon) in March 2017. The identification of plant specimens was done at the Cameroon National Herbarium in Yaoundé by Mr. Tadjouteu Fulbert in comparison with a voucher specimen under the reference number 10701/SRF. Cam.

The leaves were shade dried and ground into a fine powder. The aqueous extract was prepared by decoction. The powder (500 g) was boiled in 3.5 L of distilled water for 15 minutes. The mixture was filtrated on Whatman paper No. 1. The residue was re-extracted as previously described in 2 L of distilled water for 15 minutes. The two filtrates obtained were pooled together and evaporated in a ventilated steam room for 72 hours at 40°C to yield 89.54 g (17.91%).

The methanol extract was prepared as maceration. The powder (500 g) was soaked in 3.5 L of methanol for 72 hours and filtrated using Whatman paper No. 1. The plant material residue was re-extracted in 2 L of methanol for 24 hours. The two filtrates obtained were mixed and concentrated in a rotary evaporator under low pressure at 40°C and yielded 111.04 g (22.21%) of methanol extract.

Both aqueous and methanol extracts were prepared in 4% DMSO for the experiment prior to administration to animals.

### 2.2. Chemicals and Drugs

Uric acid, hexadecyltrimethylammonium bromide, epinephrine, O-dianisidin, and sodium azide were purchased from Sigma-Aldrich Chemical Co. (Taufkirchen, Germany). Sodium hydroxide, hydrochloric acid, bovine serum albumin, sodium chloride, and Dimethylsufoxide (DMSO) were purchased from Carl-Roth (Kalshur, Germany). Nitric acid was purchased from Qualikems. Allopurinol was obtained from Aspen-Pharma (Ireland). Ether was purchased from a local pharmacy (Dschang, Cameroon). Griess reagent, Biuret reagent, and carbonate buffer were prepared in the laboratory with ingredients from Sigma-Aldrich Chemical Co. (Taufkirchen, Germany). Thiobarbituric acid was purchased from Shanghai Zhanyun Chemical Co. Ltd (China). Trichloroacetic acid was purchased from Guangdon Guanghua Sci-Tech Co. Ltd (China).

### 2.3. Monosodium Urate (MSU) Crystal Synthesis

MSU crystals were synthesized according to a modified protocol of Tank et al. [17]. Two-hundred milliliters of an aqueous solution of NaOH (1 M) was stirred well and heated up to 70°C. After obtaining the required temperature, 1 g of uric acid was added into the NaOH solution and HCl (1N) was then used to maintain the mixture at pH 7.2. The solution was stirred at a constant temperature (70°C) for 4 hours and then stirred slowly at room temperature and stored at 4°C for 24 h. Fine particles of MSU crystals were found at the bottom of the glass. MSU particles were recovered by filtration, washed with distilled water; air-dried, and suspended in sterile saline (100 mg/ml).

### 2.4. Animals

Albino Wistar rats (150–200 g) were bred onsite in the Laboratory of Physiology and Phytopharmacology of the University of Dschang, Cameroon. Forty-two animals (10 to 12 weeks old) of either sex were divided into 7 experimental groups of 6 animals each (3 males and 3 females). Rats were habituated to pain threshold recording and paw volume measurement before the experiment. Rats with baseline pain threshold below or equal to 7.5 g were discarded.

### 2.5. MSU-Induced Gouty Arthritis

The experiment was set following the method of Zhang et al. [18], with some modifications, and performed as it is described below. Briefly, rats were anesthetized with ether, the left hind leg skin was sterilized with 75% ethanol, and the lateral malleolus located by palpation. Then, an insulin syringe needle was inserted vertically to penetrate the skin and turned distally for insertion into the articular cavity at the gap between the tibiofibular and tarsal bone until a distinct loss of resistance was felt. Fifty microliters of MSU was injected into the articulation to induce gouty arthritis (day 0). The same procedure was followed for all the groups except for the sham group in which only sterile saline was injected in the articulation. One day after MSU injection, the second baselines of inflammation and pain threshold were taken. Then, the 6 MSU-injected groups were treated as follows: negative control treated orally with DMSO (4% in distilled water), positive control treated with Allopurinol (5 mg/kg/day; dissolved in distilled water), and the 4 remaining groups treated orally with AEPP or MEPP at the doses of 100 and 200 mg/kg/day. The MSU-injected rats were treated daily for 6 consecutive days. The pain threshold at the ankle and paw was recorded using an analgesimeter (Ugo Basile, type 37215) on days 1, 2, 3, 4, 5, and 6 after the initiation of treatments. Briefly, awake rats were scruffed and placed in the analgesimeter. For each measuring time point, two trials were completed on the ipsilateral ankle and the values averaged. The same measurement procedure was conducted on the ipsilateral paw.

Immediately after pain threshold measurements, the paw and the ankle volumes were recorded using an electronic caliper (Fine Science, Heidelberg, Germany). Rats were slightly restrained, and the diameter of the paw was measured at the space between all footpads from the back (dorsal surface) to the front (plantar surface) of the paw and even the ankle.

On day 7 after MSU injection, animals were anesthetized by intraperitoneal injection of 15% ethyl carbamate solution (1.5 g/kg). Blood, spinal cord, and the left and right hemispheres were collected for antioxidant assays. The synovial liquid of the injected ankle was collected by washing the joint with 100 *μ*l of NaCl 9‰ for the evaluation of myeloperoxidase level.

Blood collected in nonheparinized tubes was centrifuged at 3000 rpm, and the serum was kept at −20°C until use. The entire spinal cord and the left and right hemisphere were homogenized in 10% and 15% solution of ice-cold 0.1 M Tris buffer (pH 7.4) at 4°C, respectively, and centrifuged at 10,000 rpm at 4°C for 15 min. The supernatant was collected and keep at −20°C. Serum, spinal cord, left and right hemisphere supernatants were used for superoxide dismutase (SOD), nitrite oxide (NO), and malondialdehyde (MDA) estimation.

### 2.6. Nitric Oxide, Superoxide Dismutase, and Malondialdehyde Assays

The Griess reagent was used to estimate the nitric oxide content. To 250 *μ*l of the tissue sample was added 250 *μ*l of 1% sulfaniladide prepared in 5% orthophosphoric acid. The mixture was incubated for 5 minutes in the dark, then 250 *μ*l of 0.1% naphthyl ethylenediamine was added, and then all incubated in the dark for additional 5 minutes. The optical density was read at 530 nm. The quantity of nitric oxide was calculated from the sodium nitrite's standard curve.

The SOD activity assessment was conducted according to the protocol of Wandji et al. [19]. To 70 *μ*l of the sample was added 830 *μ*l of carbonate buffer (pH = 10.2) and 100 *μ*l of epinephrine (0.3 mM). The mixture was homogenized, and the absorbance read 60 and 120 seconds after epinephrine was introduced, at 480 nm using a spectrophotometer (Helios Epsilon). The inhibitory percentage of oxidation was calculated according to the following formula:

(1) $$\begin{array}{c} I\%=100-\left[\frac{\text{Ab sample}}{\text{Ab blank}}\right]\times 100, \end{array}$$

where *I*% = inhibitory percentage and Ab = absorbance.

The SOD activity was calculated as:

(2) $$\begin{array}{c} A=\frac{I\%}{50\times \text{protein quantity}},\text{ with AA}=\text{the SOD activity},50=\text{one unit.} \end{array}$$

For the MDA assay, 500 *μ*l of orthophosphoric acid (1%) and 500 *μ*l of thiobarbituric acid (1%) were added to 100 *μ*l of serum/supernatant. The mixture was homogenized and placed in boiling water bath for 15 minutes. Thereafter, the tubes were cooled on ice bath and centrifuged at 3000 rpm for 10 minutes. The supernatant was collected, and the absorbance was read at 532 nm using a spectrophotometer (Helios Epsilon). The concentration of MDA was calculated based on the absorbance coefficient according to the following formula: D. O. = *ε*. C. L (where D. O. = optical density, *ε* = molar extinction coefficient of the MDA (1.56 × 10^5^ M^−1^. Cm^−1^), *C* = concentration in MDA, and *L* = length of the optic journey (1 cm)).

### 2.7. Myeloperoxidase Assay

This assay was conducted as previously described by Garça et al. [20], with some modifications. In each well of a 96-well plate, 25 *μ*l of the synovial liquid, 25 *μ*l of potassium phosphate buffer, and 25 *μ*l of 0.5% hexadecyltrimethylammonium bromide buffer were introduced. The mixture was incubated for 30 minutes at room temperature. Then, 10 *μ*l of sodium azide (0.1 mM) and 150 *μ*l of potassium phosphate buffer (50 mM; pH 5.5) containing 0.026% ortho-dianisidine dihydrochloride and 0.018% hydrogen peroxide were added to the reaction medium. The entire plate was once again incubated at room temperature for 30 minutes. Finally, the optical density of each well of the 96-well plate was read using an ELIZA microplate reader at 450 nm. MPO level was expressed using an arbitrary unit.

### 2.8. Statistical Analysis

Results are expressed as mean ± SEM. Time-dependent hyperalgesia and inflammation were analyzed using two-way ANOVA followed by Bonferroni's posttest while time point data were analyzed with one-way ANOVA followed by Tukey's posttest. All the analyses were performed with GraphPad Prism 5.01 software package. A *p* value of less than 0.05 was considered as statistically significant.

## 3. Results

### 3.1. Anti-Inflammatory Effects of *P. pinnata* Extracts on MSU-Induced Gouty

**Figure 1:**
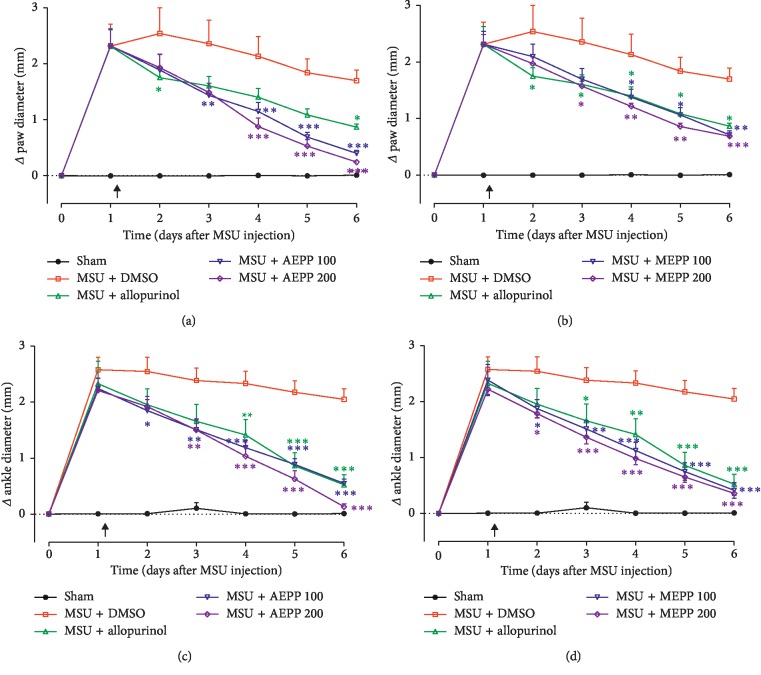
Effects of the (a, c) aqueous (AEPP 100, 200 mg/kg/day) and (b, d) methanolic (MEPP 100, 200 mg/kg/day) extracts of *Paullinia pinnata* on the primary (ankle (a, b)) and secondary (paw (c, d)) inflammatory gout induced by MSU ankle injection in rat. (a) AEPP induces significant decreases in ankle swelling compared to rats treated with MSU and vehicle (3%DMSO). (b) MEPP induces significant decreases in ankle swelling compared to rats treated with MSU and vehicle. (c) AEPP induces significant decreases in paw swelling compared to rats treated with MSU and vehicle. (d) MEPP induces significant decreases in paw swelling compared to rats treated with MSU and vehicle. Allopurinol (5 mg/kg/day) is included as a positive control for anti-inflammatory effects. Each point represents the mean ± SEM of 6 individual rats. ^*∗∗*^*p* < 0.01, ^*∗∗∗*^*p* < 0.001; significant difference compared to the negative control group (MSU + DMSO) using two-way ANOVA with Bonferroni posttest. The black arrow indicates the beginning of treatments.

Intra-ankle injection of MSU resulted in a significant inflammation that was maintained for at least 6 days. Daily oral administration of *P. pinnata* extracts significantly reduced the inflammation induced by MSU both at the level of the ankle and the paw. Two-way ANOVA (main effects time, treatment, and interaction) demonstrated significant effects at all the doses used (*p* < 0.05; *p* < 0.01; *p* < 0.001) (Figures 1(a)–1(d)). Roughly, both AEPP and MEPP showed dose-dependent effects. At the dose of 200 mg/kg, AEPP and MEPP exhibited maximal inhibitory percentages of 99.23 and 98.40%, respectively. The inhibitory percentage induced by allopurinol (5 mg/kg/day) ranged from 26.76 to 90.81% and was, in general, less efficient than *P. pinnata* extracts (Figure 1).

### 3.2. Antihyperalgesic Effects of *P. pinnata* Extracts on MSU-Induced Gouty

**Figure 2:**
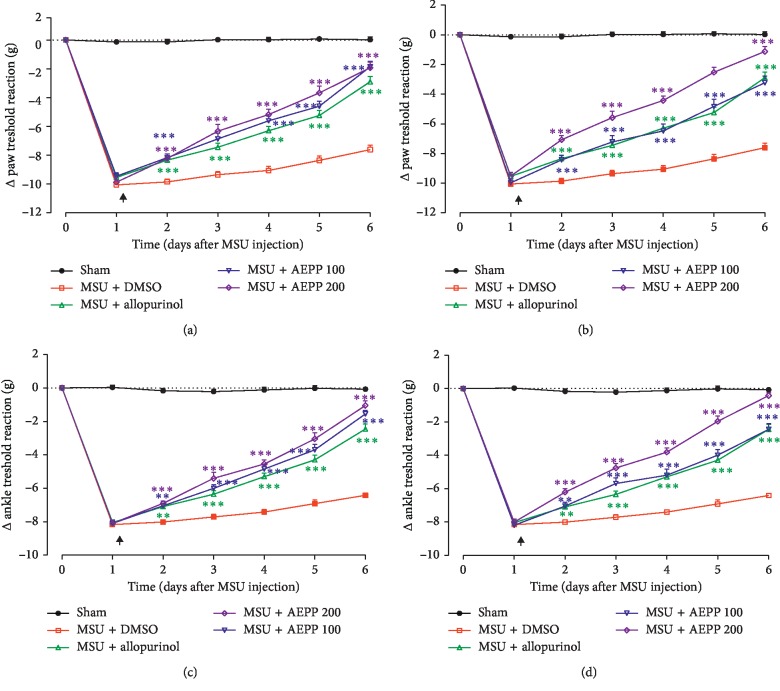
Effects of the aqueous (AEPP; 100, 200 mg/kg/day) and methanol (MEPP; 100, 200 mg/kg/day) extracts of *Paullinia pinnata* on the primary (ankle) and secondary (paw) mechanical hypersensitivity induced by ankle injection of MSU in rat. (a) AEPP induces significant decreases in mechanical hypersensitivity (i.e., decreased change in threshold from baseline) compared to rats treated with MSU and vehicle (3%DMSO). (b) MEPP induces significant decreases in mechanical hypersensitivity compared to rats treated with MSU and vehicle. (c) AEPP induces significant decreases in mechanical hypersensitivity compared to rats treated with MSU and vehicle. (d) MEPP induces significant decreases in mechanical hypersensitivity compared to rats treated with MSU and vehicle. Allopurinol (5 mg/kg/day) is included as a positive control for the antihyperalgesic effect. Each point represents the mean ± SEM of 6 individual rats. ^*∗∗*^*p* < 0.01, ^*∗∗∗*^*p* < 0.001; significant difference compared to the negative control group (MSU + DMSO) using two-way ANOVA with Bonferroni posttest. The black arrow indicates the beginning of treatments.

The intra-articular administration of MSU resulted in hyperalgesia both at the level of the ankle and the paw. The oral administration of *P. pinnata* extracts starting one day after MSU injection significantly reduced the mechanical hyperalgesia as measured with the Randall Sellito method (two-way ANOVA effects time, treatment, and interaction *p* < 0.01; *p* < 0.001). As demonstrated by Bonferroni posttest, AEPP, and MEPP induced time- and dose-dependent antihyperalgesic effects (Figures 2(a)–2(d)). MEPP at the dose of 200 mg/kg almost completely abolished the mechanical hyperalgesia, both at the level of the paw and the ankle (Figures 2(b) and 2(d)). The effect of allopurinol was equal to that of MEPP at the dose of 100 mg/kg.

### 3.3. Inhibitory Effects of *P. pinnata* Extracts on the MPO Activity in Synovial Liquid in Gouty Rats

**Figure 3:**
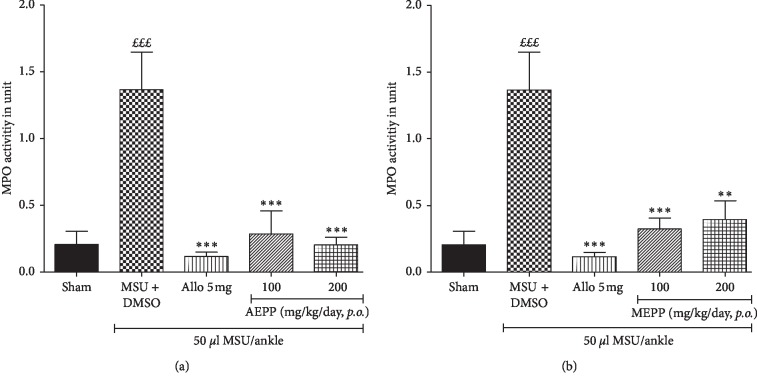
Effects of the aqueous (AEPP) and methanol (MEPP) extracts of *Paullinia pinnata* on myeloperoxidase (MPO) activity in the synovial liquid of MSU-injected animals. Allopurinol (5 mg/kg/day) was used as a positive control. (a) AEPP treatment at 100 mg/kg and 200 mg/kg reversed MSU-induced increases in MPO activity in synovial liquid. (b) MEPP treatment at 100 and 200 mg/kg reversed MSU-induced increases in MPO activity in synovial liquid. Each bar represents the mean ± SEM of 6 individual rats. ^*∗∗*^*p* < 0.01, ^*∗∗∗*^*p* < 0.001; significant difference compared to the negative control group (MSU + DMSO) using one-way ANOVA with Tukey's posttest. ^£££^*p* < 0.001 significant difference compared to the sham group using one-way ANOVA with Tukey's posttest.

In the process of inflammation, neutrophils through their oxidative burst produce MPO. As shown in Figure 3, the intra-articular injection on MSU drastically increased (*p* < 0.001) the MPO activity in the synovial fluid as compared to the sham group. AEPP and MEPP significantly (*p* < 0.01; *p* < 0.001) reduced the activity of MPO, with the highest effect observed with AEPP at the dose of 200 mg/kg (Figure 3(a)). The positive control allopurinol-treated group (5 mg/kg) (Figures 3(a) and 3(b)) also significantly (*p* < 0.001) reduces the MPO level as compared to untreated MSU control and more efficiently than both AEPP and MEPP at both doses (100 and 200 mg/kg).

### 3.4. *P. pinnata* Extracts Inhibit the NO Production in the Spinal Cord and the Right and Left Hemispheres of Gouty Rat

**Figure 4:**
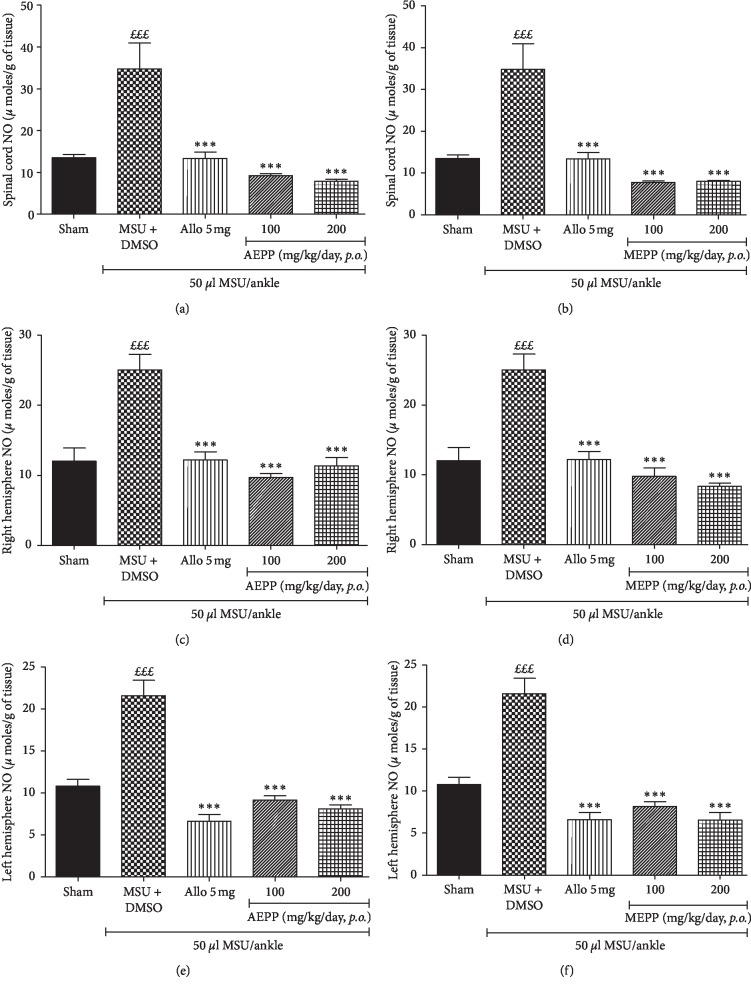
Effects of the aqueous (AEPP) and methanolic (MEPP) extracts of *Paullinia pinnata* on the nitrite oxide level in the spinal cord (a, b), the right hemisphere (c, d), and the left hemisphere (e, f). Plant extract treatment in all the organs used significantly reversed the overproduction of nitric oxide (NO) induced by the intra-ankle injection of MSU. Each bar represents the mean ± SEM of five repetitions. ^*∗∗∗*^*p* < 0.001 significant difference compared to the negative control group (MSU + DMSO) using one-way ANOVA with Tukey's posttest. ^£££^*p* < 0.001 significant difference compared to the sham group using one-way ANOVA with Tukey's posttest.

During the inflammatory process, immune cells play an essential role while defending the organism by producing nitrite oxide. For more insight on the anti-inflammatory activities of *P. pinnata* extracts, we evaluated the effect of AEPP and MEPP on the nitric oxide production in the spinal cord and the right and left hemispheres. The control group (MSU-DMSO) showed a marked increase (*p* < 0.001) in the NO content in all the sample regions collected. This increase was significantly and completely reversed (*p* < 0.001) by *P. pinnata* extracts and allopurinol in all the collected organs and at all the doses used, as compared to the control group (Figures 4(a)–4(f)).

### 3.5. Effects of *P. pinnata* on the SOD Activity in the Serum, Spinal Cord, and Right and Left Hemispheres of Gouty Rat

**Figure 5:**
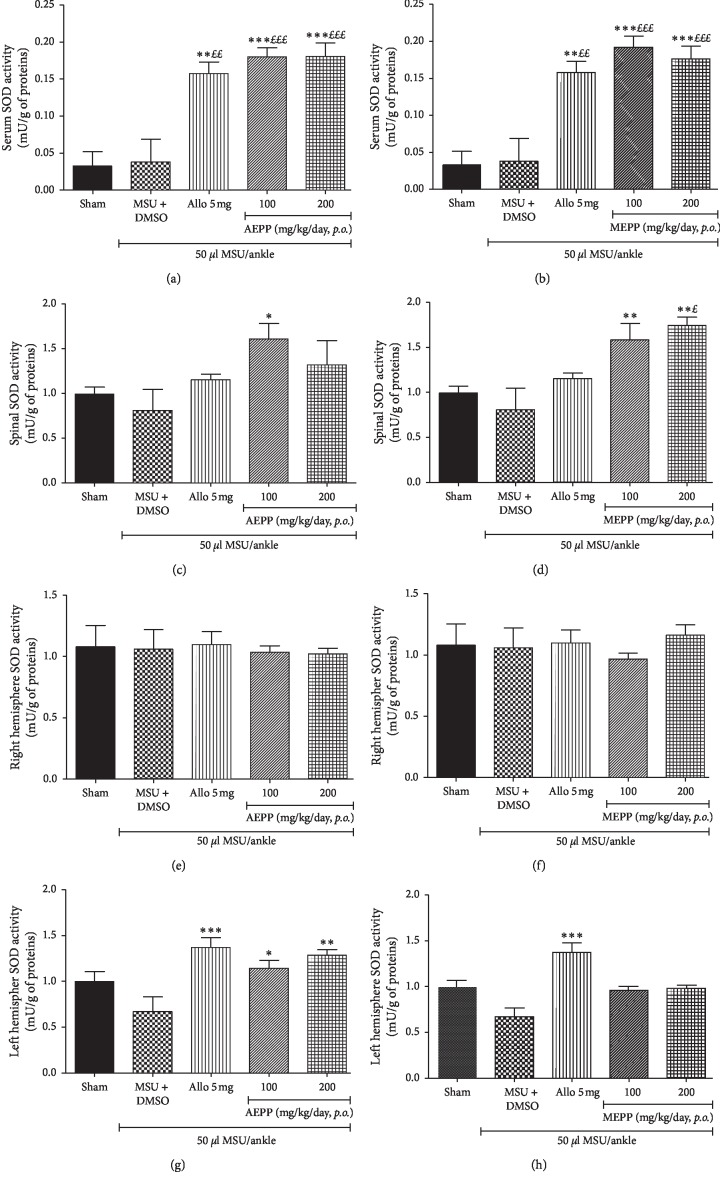
Effects of the aqueous (AEPP) and methanol (MEPP) extracts of *Paullinia pinnata* on the superoxide dismutase (SOD) activity in the serum (Figures 4(a) and 4(b)), spinal cord (Figures 4(c) and 4(d)), right hemisphere (Figures 5(e and f)), and left hemisphere (Figures 5(g and h)) of MSU-injected rats. Allopurinol (5 mg/kg/day) serves as a positive control for antigouty effects. SOD activity significantly increased after treatment with allopurinol, AEPP (100 and 200), and MEPP (100 and 200) in the serum (a and b). Only the AEPP 100 and the MEPP (100 and 200) significantly increase SOD activity in the spinal cord (c and d). Both the AEPP (100 and 200) and the MEPP (100 and 200) fail to increase SOD activity in the right hemisphere (e and f). The AEPP 200 and allopurinol significantly reverse MSU-induced SOD exhaustion in the left hemisphere (g and h). Each bar represents the mean ± SEM of 6 individual rats, ^*∗*^*p* < 0.05, ^*∗∗*^*p* < 0.01, ^*∗∗∗*^*p* < 0.001; significant difference compared to the negative control group (MSU + DMSO) using one-way ANOVA with Tukey's posttest. ^£^*p* < 0.05, ^££^*p* < 0.01, ^£££^*p* < 0.001; significant difference compared to the sham group using one-way ANOVA with Tukey's posttest.

The impact of *P. pinnata* extracts on oxidative stress was evaluated through the SOD activity measured in the serum, spinal cord, and right and left hemispheres. MSU ankle injection did not significantly affect the SOD activity in all the tissues used, as compared to the sham group. However, the daily administration of AEPP and MEPP induced a tissue-dependent increase in the SOD activity. In fact, *P. pinnata* extracts did not affect the evaluated parameter in the right hemisphere. Besides, MEPP showed no effect on the SOD activity in the left hemisphere (Figure 5(h)). Allopurinol was able to significantly increase the SOD activity only in the serum and the left hemisphere, while both extracts did so in the serum and the spinal cord. More, AEPP increases the parameter in the left hemisphere in a dose-dependent manner (Figure 5(g)).

### 3.6. *P. pinnata* Extracts Reduce the MDA Content in the Spinal Cord and Right and Left Hemispheres of Gouty Rat

**Figure 6:**
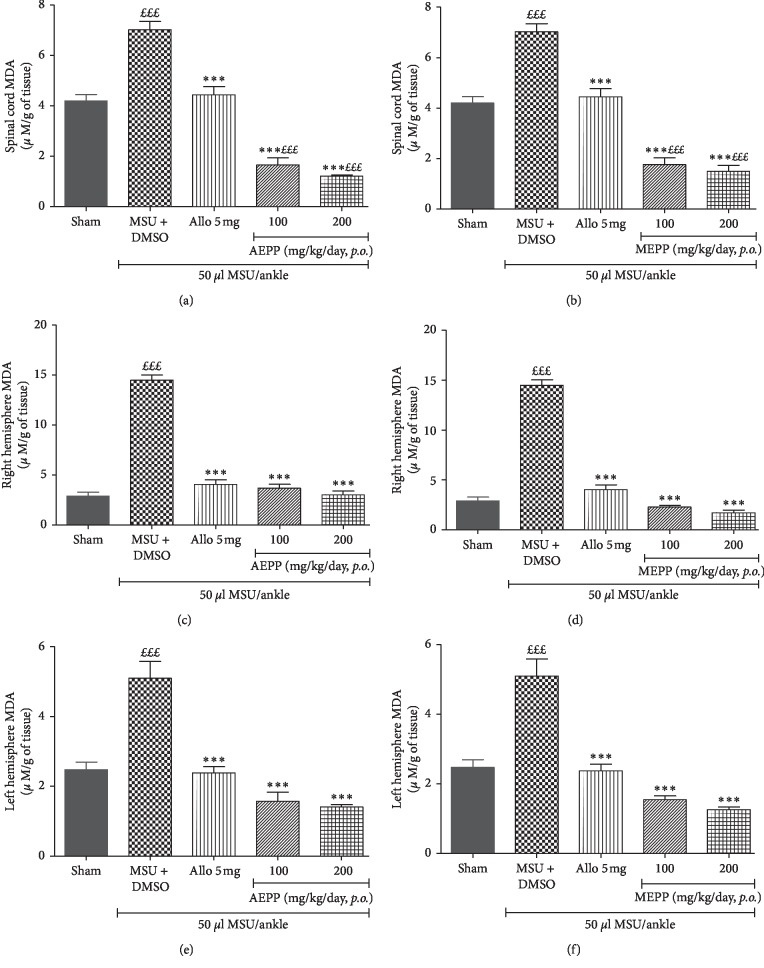
Effects of the aqueous (AEPP) and methanol (MEPP) extracts of *Paullinia pinnata* on the malondialdehyde (MDA) content in the spinal cord of MSU-injected animals. Allopurinol (5 mg/kg/day) is used as a positive control for antigouty effects. (a) AEPP treatment at 200 mg/kg and 300 mg/kg reversed MSU-induced increases in MDA spinal cord content (one-way ANOVA significant main effect *p* < 0.001). (b) MEPP treatment at 100, 200, and 300 mg/kg reversed MSU-induced increase in MDA spinal cord content (one-way ANOVA significant main effect *p* < 0.001). Each bar represents the mean ± SEM of 6 individual rats. ^*∗∗∗*^*p* < 0.001; significant difference compared to the negative control group (MSU + DMSO) using one-way ANOVA with Tukey's posttest. ^£^*p* < 0.05, ^££^*p* < 0.01, ^£££^*p* < 0.001; significant difference compared to sham using one-way ANOVA with Tukey's posttest.

MDA is the end-product of lipid peroxidation and can indicate the status of oxidative stress. To evaluate the protective effect of *P. pinnata* extracts against the oxidative damages in the pain modulating/integrative structures, we quantified the MDA content in the spinal cord and cerebral hemispheres. As expected, we found a significant (*p* < 0.001) increase of MDA content in MSU-injected rats as compared to the sham group (Figures 6(a)–6(f)). AEPP and MEPP significantly (*p* < 0.001) reduced the MDA content in the spinal cord and right and left hemispheres as compared to the MSU-control group. The positive control allopurinol also produces a significant (*p* < 0.001) effect, but this inhibitory activity was lower than that of both extracts independently of the tissue.

## 4. Discussion

The present study was undertaken to investigate the ability of the aqueous (AEPP) and methanol (MEPP) extracts of *P. pinnata* against pain, inflammation, and oxidative stress in gouty arthritis, a disease of the musculoskeletal system characterized by painful inflammatory joints and oxidatives. Gout is the result of the accumulation of monosodium urate (MSU) crystals in the joint. Thus, experimental MSU-induced gouty arthritis is a trustworthy model of gout that mimics the clinical human condition. In the present study, the intra-ankle injection of MSU resulted in inflammation and hyperalgesia in both the ipsilateral ankle and paw, accompanied by an increase in MPO activity in synovial fluid and oxidative stress in the spinal cord and the brain. These results largely corroborate the previous findings [21–23].

Oral administration of the AEPP and MEPP significantly reduced inflammation and hyperalgesia in a dose- and time-dependent manner, reduced the myeloperoxidase activity in synovial fluid, and exhibited significant in vivo antioxidant effects on NO, MDA, and SOD.

Cumulative literature is concordant on the physiopathological process of MSU-induced gout. In fact, at the first step, phagocytes in the joint such as macrophages, monocytes, or neutrophils will activate upon detection of MSU crystals, to produce active IL-1*β* which will subsequently activate NF-КB to trigger the transcription of cytokines and chemokines as well as the release of inflammatory mediators. The released mediators will finally activate nociceptor endings, opening TRPV1 and TRPA1 channels either directly or through intracellular signaling cascades. Furthermore, the antidromic firing of articular nociceptors releases neuropeptides from peripheral nerve terminals, thereby potentiating the local inflammatory response known as neurogenic inflammation [24, 25]. As a consequence of these intricate pathways, there will be hypersensitization of nociceptors ending for hypernociception and allodynia, as well as inflammation in the joint and surrounding tissues. AEPP and MEPP showed time-dependent analgesic and anti-inflammatory effects, suggesting that these extracts possess bioactive molecules that may interfere with the different pathways described above. *P. pinnata* extracts may, therefore, act on phagocytes and directly on the nervous system. Previous studies with the same extracts of *P. pinnata* demonstrated their analgesic and anti-inflammatory effects in Complete Freund's Adjuvant- (CFA-) induced monoarthritis and suggested that the extracts may have effect both at the central and peripheral levels [16]. Besides, it was also demonstrated that these extracts inhibit the production of IL-1*β* and TNF-*α* by macrophages in inflammatory conditions. As IL-1*β* plays a pivotal role in the development of pain and inflammation in the context of hyperuricemia or MSU accumulation in the joint, it can be suggested that the analgesic and anti-inflammatory effects of *P. pinnata* extracts are related to their IL-1*β* inhibition capacity. As such, the analgesic effect of the plant extracts will be highly dependent on its anti-inflammatory properties.

Nevertheless, AEPP and MEPP were able to reduce the secondary analgesia at the level of the paw. This result suggests that *P. pinnata* extracts may act directly on the central nervous system, as secondary pain depends mostly on the sensitization of the central nervous system [26].

It has clearly been shown that neutrophil recruitment and activation are the cornerstones of the inflammatory response to monosodium urate (MSU) crystals [27]. Neutrophils infiltrated and accumulated in the synovial joint will undergo degranulation and release their secretion products among which are myeloperoxidases (MPO). To determine the effects of *P. pinnata* extract on neutrophils infiltration and activation, we evaluated the MPO activity in the synovial fluid. Both AEPP and MEPP potently reduced the MPO activity, confirming that these extracts reduce cell infiltration as previously demonstrated [16]. Also, the possibility that *P. pinnata* extracts could inhibit the release of inflammatory mediators from leucocytes cannot be rolled out, as previously discussed. It has been shown that the methanol extract of the leaves of *P. pinnata* possesses *β*-sitosterol and friedelin [14]. The analgesic and the anti-inflammatory properties of these compounds have been demonstrated [28, 29]. Therefore, it can be assumed that the analgesic and anti-inflammatory effects of the methanol extract of *P. pinnata* is related to the presence of *β*-sitosterol and friedelin.

One of the early events associated with gout is the oxidative stress [30] at the level of the affected joint. Besides, the constant transmission of noxious stimulus in the spinal cord and the brain, promotes the release of reactive oxygen species (ROS), leading to oxidative stress in these tissues. It has been demonstrated that ROS mediate the development and maintenance of pain and further boost the peripheral and central sensitization in joint pain [30]. Concordantly, it was observed in the present study that MSU-induced gout was accompanied by substantial production of NO and malondialdehyde in the spinal cord and the brain while the superoxide dismutase content was not significantly affected. These data confirm the oxidative status in the brain and spinal cord of MSU gouty animals. AEPP and MEPP significantly reduced both NO and MDA contents and increased the SOD activity in the tissues, further confirming the in vivo antioxidant effects of *P. pinnata* extracts as previously suggested [16].

## 5. Conclusion

The present study shows that the aqueous and methanol extracts from the leaves of *P. pinnata* possess antihyperalgesic and anti-inflammatory effects against MSU-induced gouty arthritis. The analgesic effect is at least partially related to its anti-inflammatory activity. The anti-inflammatory effect seems to be due to the inhibition of the inflammatory cells infiltration and/or the inactivation of infiltrated cells. The antioxidant effect of these extracts also highly contributes to their antigouty activity. These results support the use of the leaves of *P. pinnata* extracts in Cameroonian folk medicine for the management of gouty arthritis.

## Data Availability

The data used to support the findings of this study are available from the corresponding author upon request.

## Abbreviation

MSU: Monosodium urate
AEPP: Aqueous extract *Paullinia pinnata*
MEPP: Methanol extract *Paullinia pinnata*
NaOH: Sodium hydroxide
HCl: Hydrogen chloride
DMSO: Dimethyl-sulfoxide
MDA: Malondialdehyde
NO: Nitrite oxide
SOD: Superoxide dismutase
MPO: Myeloperoxidase
ROS: Reactive oxygen species
CFA: Complete Freud's Adjuvant.
